# Self-management interventions for common long-term conditions in low- and middle-income countries: a synthesis of current evidence

**DOI:** 10.7189/jogh.15.04148

**Published:** 2025-05-23

**Authors:** Taim Akhal, Margherita Gabra, Miriam Adesokan, Opeyemi O Babatunde

**Affiliations:** 1 The West African Institute for Applied Health Research (WAFERs), Ibadan, Oyo State, Nigeria; 2 Keele University, School of Medicine, Keele, Newcastle-under-Lyme, Staffordshire, UK

## Abstract

**Background:**

In addition to endemic communicable diseases, resource-poor health systems in low- and middle-income countries (LMICs) are buckling under an increasing prevalence, morbidity, and mortality attributable to non-communicable diseases (NCD) – ‘NCDs crisis.’ Supported self-management (SM) is a recommended approach for improving patient outcomes. There is yet no robust evidence on the effectiveness of SM interventions for people with long-term conditions (LTCs) in LMICs.

**Methods:**

Underpinned by a comprehensive search of seven databases, we conducted a systematic review and evidence synthesis. Paired reviewers completed study selection, methodological, and data extraction. Here, we report a synthesis considering disease-specific (*e.g.* glycated haemoglobin (HbA1c), quality-of-life (QoL), and economic outcomes.

**Results:**

Of 49 222 citations, 26 studies were included in the analysis (one systematic review, 14 trials, five cross-sectional, and six qualitative studies). LTCs for which SM interventions were reported included: diabetes (14 studies, n = 2388), hypertension (six studies, n = 1779), and arthritis (two studies, n = 180). Further, three studies (n = 237) were on co-morbid diabetes and hypertension. Interventions were broadly classified as: SM education, mobile health-guided SM, and community-based support for SM. Education on SM showed the most promising improvement in clinical outcomes (*e.g.* mean pain intensity scores from 3.97 to 2.77), functional ability, HbA1c (pre-intervention mean of 8.58% to a post-intervention 8.08% in one study; 9.45% to 8.98% in another study), millimetres of mercury (mmHg) (pre-intervention mean of 129.7/83.7 to a post-intervention 117.9/75.3) and health-related QoL (*e.g.* EuroQol Five Dimension score improvement from 0.77 to 0.89 post-intervention) among patients living with diabetes, hypertension and arthritis compared to usual care. Effectiveness of interventions was dependent on literacy, SM education delivery aids, and disease-specific care, as patients preferred in-person (interactive) education sessions over virtual assistance alone.

**Conclusion:**

Guided SM interventions with community-based support show promise for improving outcomes for people with common LTCs in LMICs. However, a dearth of cost-feasibility data and variability in outcomes limit decisions on scalability and policy decision making. There is a need for regulatory bodies to develop clinical guidelines and promote implementation of tailored SM education as a core management strategy for LTCs care in LMICs.

**Registration:**

PROSPERO CRD42022345762.

The World Health Organization (WHO) and other literatures reports increasing morbidity and mortality from chronic non-communicable diseases (NCDs) with 86% of premature deaths occurring in low- and middle-income countries (LMICs) [1–3]. Non-communicable diseases are projected to rise sharply in LMICs, at a greater rate and impact than in high-income countries (HICs) [1–4]. According to the World Bank, NCDs increasingly threaten the health care and economy of LMICs, with these systems not being adequately prepared to face the NCD crisis [5,6]. However, policymakers, donors and academics have consistently directed more efforts towards acute infectious disease control in resource-limited settings [1]. Unfortunately, ‘lengthy and expensive’ treatment costs for NCDs also known as long-term conditions (LTCs) quickly drain household resources and force people in LMICs deeper into poverty, increasing their risk of worsened morbidity and mortality [2].

Self-management typically involving individualised partnership with health care providers where the patient actively takes part in their care, are recommended first-line interventions in HICs [7–10]; but these are highly dependent on individual health literacy, and self-efficacy, and are yet to be fully explored in LMICs context for people living with LTCs. Health is a collective and collaborative investment and patients have a crucial role in the management of their disease. During this process, patient develops knowledge on their condition and assume personal skills and coping mechanisms to deal with disease-related adversity [11]. This fosters a care culture that supports patients as partners in co-creating health, embeds person-centred care in everyday life within the community, ultimately optimises health outcomes, and reduces the burden and need for direct constant care from health care professionals (HCPs) in hospitals. Empowering people through self-management is an excellent way of improving overall patient health, as well as relieving economic stress on the public and overstretched health care systems of LMICs.

Current evidence support the effectiveness and patient benefit from self-management interventions for LTCs in HICs [7–10] in form of moderate to strong association with improved clinical outcomes [12], increased engagement in self-care behaviour [7], effective mental illness management [8], and overall positive health outcomes [9,10]. However, there is yet no robust synthesised evidence on self-management of LTCs in LMICs. A scoping review highlighted emerging evidence base in support of the employability of self-management for improving care in LMICs [13], but it was limited to diabetes and cardiovascular conditions and did not involve an appraisal of methodological quality which is crucial to guide policy and inform clinical decision making. This review aims to present a more up-to-date and appraised evidence via the inclusion of newer studies, other common LTCs (including osteoarthritis and mental health conditions), and considerations for self-management in co-morbid LTCs. The overall aim of this study was to summarise and critically appraise currently available evidence regarding self-management of common LTCs in LMICs. Specific objectives were to identify and appraise current evidence on interventions designed to facilitate self-management of common LTCs in LMICs context, in terms of clinical, quality of life and economic outcomes and explore what are the barriers and facilitators to effective self-management of LTCs in LMICs.

## METHODS

### Protocol and registration

We established a research protocol prior to conducting review. It was submitted to the International Prospective Register of Systematic Reviews (PROSPERO-ID CRD42022345762). We reported this review in conformity with the PRISMA statement [14].

### Information sources and search strategy

We developed, in collaboration with the information specialist, a search strategy for relevant studies relating to LTCs, and self-management in LMICs. We conducted a comprehensive search of databases (MEDLINE, EMBASE, CINAHL, PsycINFO, AMED, Cochrane Library, and Web of Science) for relevant primary studies without setting any date limits up until June 2022. The detailed search strategy (*e.g.* MEDLINE) is provided in Appendix S1 in the **Online Supplementary Document**. In addition, we examined reference lists of included articles to identify other potentially relevant article(s). We managed literature search results in Rayyan (Rayyan Systems Inc., Boston, Massachusetts, USA), an online screening software.

### Eligibility and study selection

We developed and agreed upon the detailed eligibility criteria. Briefly, we included studies if they reported original research on self-management interventions of one or multiple LTCs, and included adults aged ≥45 years residing in LMICs. We selected the age criteria based on documented age of onset of most common LTCs [15–20] and pragmatically based on contextual knowledge that approach to management, organisation of care, and access to care are also likely to differ for older adults across many health infrastructures in LMICs.

We defined LMICs according to the World Bank’s country and lending group list and excluded studies conducted in HICs. We prioritised interventions that encouraged patients to self-manage at home or in community settings, rather than self-management programmes that are fully supervised by trained health service providers or take place mostly in health care facilities. To be eligible, self-management typically involves individualised or collective partnership with health care providers where the patient actively takes part in their care.

To be eligible, participants needed to be diagnosed with any one or more common LTCs [20,21] including diabetes mellitus, hypertension, musculoskeletal conditions (*e.g.* joint pain including arthritis of the knee, hip, back, and widespread pain), which are high contributors to disability-adjusted life years [3] in LMICs. Co-morbid mental health problems, such as depression and anxiety, were also eligible, given emerging evidence that mental health problems often co-occur with physical health issues [20].

To gain a comprehensive overview of all currently available evidence, the included study designs were either experimental (*e.g.* randomised trials, comparative cohort studies, before-after designs) or non-experimental (prospective or retrospective observational cohort studies, qualitative studies, cross-sectional surveys). We excluded case studies.

There were no restrictions on the language of publication or the date of publication. However, primary studies published before June 2020, investigating self-management interventions for diabetes in LMICs were excluded, due to the existence of a comprehensive systematic review [22] of self-management in diabetes up to June 2020. It was necessary to apply this criterion in accordance with good practice in order to avoid duplication and research waste. We have therefore included summary information from this review as part of the evidence on self-management intervention for diabetes in LMICs.

An initial pilot screen was conducted where reviewers independently reviewed a sample of 200 titles and abstracts, and conflicts were discussed among the team to ensure agreement on the selection process and an established eligibility criterion. Reviewers subsequently screened the remaining titles and abstracts according to specified eligibility criteria. All potentially eligible full-text articles were independently reviewed by reviewers in pairs, including detailed notes/reasons for exclusion as appropriate. Disagreements on the eligibility of full texts were resolved by discussion till a consensus was reached among the reviewers or by the independent opinion of a third reviewer.

### Data items and data collection process

We used a pre-piloted and customised spreadsheet to aid data extraction from included studies. Data items included study characteristics (first author, year of publication), setting, study population characteristics, intervention characteristics (what and how interventions are delivered), treatment effectiveness and outcomes. Where available, information regarding facilitators and barriers, fidelity and feasibility of interventions, as well as patient satisfaction outcomes, was documented. Data was extracted for each paper by one reviewer (TA, MG, and MA) and was independently checked for validity and accuracy by a second reviewer (TA and OB). Disagreements were resolved by discussion until a consensus was reached.

### Quality assessment

The methodological quality of the included studies was evaluated through the Mixed Method Appraisal Tool (MMAT) [23]. The MMAT was designed to assess an array of study designs (qualitative, quantitative and mixed methods studies); and is suitable for use in complex systematic reviews that are reporting on context-dependent designs and outcomes, such as in the present study focusing on various self-management interventions across multiple LTCs in variable LMIC settings. MMAT tool assesses validity of study design (clearly formulated research question, appropriateness of outcomes, participants recruitment and selection). It also assesses aspects of study methodology such as delivery of intervention (where relevant), influence of participant characteristics on study outcomes, data analysis and study reporting. Individual MMAT items were reported as a yes, as a no or as unclear. A ‘yes’ meant the criteria were clearly met, a ‘no’ meant the criteria were clearly unmet, and an ‘unclear’ meant that the information provided by the authors was insufficient to make a judgement. This reporting was done on the individual study level and across all the included studies in this review. Differences in opinion were resolved by discussion until consensus was reached or by the independent review of a third party, who also checked for consistency. No studies were excluded on the basis of low scores on the quality appraisal tool used in this review. However, the methodological quality of the evidence base contributing to each intervention was considered in assessing the strength of evidence.

### Data synthesis and analysis

We did not conduct a meta-analysis due to a high degree of variability in outcome data across included studies. We used a narrative best evidence synthesis approach to analyse data and summarise findings. Primary analyses focused on assessing the effectiveness of self-management interventions on health-related outcomes (disease-specific, quality of life) and, where possible, economic costs/affordability. Further, primary analysis focused on the barriers/facilitators of self-management intervention and scalability in low-resource settings.

We analysed data and reported findings according to LTC categories – diabetes, hypertension, musculoskeletal pain conditions, and mental health. We explored the robustness and impact of methodological quality on overall results, mainly the effectiveness of self-management interventions. We explored subgroup analysis according to self-management intervention approach/subtypes. Variability in outcomes was also explored with reference to the impact of factors such as cultural sensitivity to approach/delivery of self-management interventions, socioeconomic status/literacy of study participants, contribution of caregivers (*e.g.* spouse) and level of involvement of health care professionals in delivery of self-management interventions. We constructed evidence tables to aid the presentation of findings.

## RESULTS

### Study flow and characteristics of included studies

**Figure 1:**
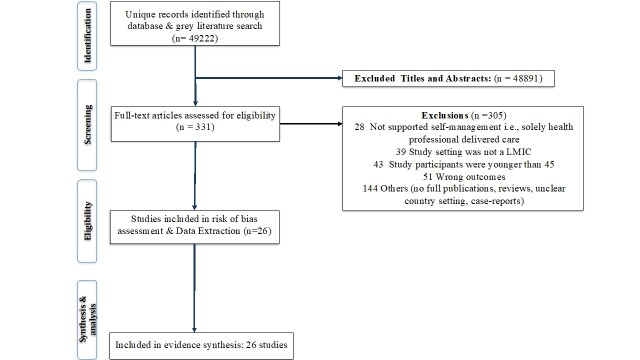
Study flow diagram.

The literature search yielded 49 222 studies, and we selected 331 studies for full-text review. Of the 331 texts, 26 studies met the inclusion criteria and were subject to data extraction and quality assessment. Key reasons for exclusion were that study participants were, on average, aged <45 years, not resident in an LMICs, interventions were solely delivered by health professionals, or studies did not report any direct outcome of self-management intervention (**Figure 1**).

In addition to one systematic review [22] and 12 randomised controlled trials (RCTs) [24–35], the review included quasi-experimental studies (n = 2) [36,37] of self-management interventions, cross-sectional (n = 5) [38–42], and qualitative studies (n = 6) [43–48] which explored patients’ experiences of self-management for LTCs. Common LTCs reported across the included studies were diabetes (n = 15) [22,24,25,28–31,36,38,39,41,45–47], hypertension (n = 6) [27,33,37,40,42,43], arthritis/musculoskeletal pain (n = 2) [33,35], co-morbid diabetes and hypertension (n = 3) [26,32,48]. Still, there were no reports of self-management for mental health conditions. In total, this study synthesises evidence of self-management of LTCs from 4570 patients across the included primary studies. Published between 2011–22, up to 40% of these studies (n = 9) [26,27,30,40-42,45–47] were from East Asia and Pacific region, while six [25,28,33,36–38] were conducted in North Africa and Middle East, five [31,35,43,44,48] in sub-Saharan Africa, four [24,29,32,39] in South Asia and one [34] in Eastern Europe (Ukraine) according to the World Bank’s geographic classification. Studies recruited participants mostly from the community or primary care settings, and only one study [29] was from a hospital outpatient clinic (**Table 1**).

**Table 1:** Characteristics of included studies

Author	Country	Year	Study type	Sample (n)	Participants average age	Female (%)	Self-management interventions/description	Primary diagnosis	Comorbidities	Type of support	Conclusion/outcomes
Almomani et al. [38]	Jordan	2021	Cross-sectional	520	55.9	49	Exercise, diet, blood glucose testing, foot care, and medication education.	T2DM	52% (hypertension, cardiac disease, renal impairments, vision impairments and peripheral neuropathy)	Caregiver	79.2% of patients had unsatisfactory self-care. Self-care and lower HbA1c are correlated. Different areas of self-care show varying compliance (exercise, education, sugar testing least; foot care and diet middle; medication most). Socioeconomic characteristics are key considerations in glycaemic control.
Rahmatullah et al. [39]	Pakistan	2021	Cross-sectional	205	52.66	37.1	Use of oral hypoglycaemic agents and insulin, self-check of blood glucose, physical activity, and daily foot care education.	T2DM	NA	None	Not following self-management practices can affect HbA1c and all diabetes related diseases, such as foot ulcers. Many reasons (lack of time and knowledge, cost, and fear) can cause non-compliance with self-management.
Nazir et al. [24]	Pakistan	2020	RCT	392	30–40 (17.6%), 41–50 (33.4%), 51–60 (34.9%), 61–70 (14.0%)	43.3	Patient education, lifestyle education and medication counselling.	T2DM	NA	Healthcare provider	Educational intervention improves diabetes knowledge, adherence, health-related quality of life and HbA1c levels.
Farag Mohamed et al. [25]	Egypt	2021	RCT	100	40–60	57	Patient education (includes nutrition, activities, smoking cessation), review of medication and visits to pharmacists.	T2DM	Hypertension	Healthcare provider	Non-pharmacological measures have an important effect on diabetes control and HbA1c reduction. A reduction in cost can be witnessed by this intervention.
Qu et al. [40]	China	2019	Cross-sectional	873	65	58.76	Diet, exercise, lifestyle management and risk factor management education.	Hypertension/co-morbidities	54.1% had one or more comorbidities: 10.4% diabetes, 7% arthritis, 6.3% COPD, 1.9% myocardial infarction, 1.3% nephropathy, 1.1% gastrointestinal disease	NA	Longer disease duration and bad insurance were associated with poor blood pressure control. Gender-related factors are recommended to be considered in order to improve self-management. Better self-management significantly decreased the odds of poor blood control.
Emara et al. [36]	Egypt	2021	Quasi-experimental study	120	47	46	Patient education on treatment, diet, exercise, medication, blood glucose monitoring, complications, and symptom management.	T2DM	Some had neurological conditions, dyslipidaemia, and hypertension	Healthcare provider	Diabetes self-management education programmes benefit glycaemic control.
Pamungkas et al. [26]	Indonesia	2022	RCT	60	56.2	80	Blood glucose monitoring, diet, online consultation, articles to educate users, app coaching, user guides, mindfulness coaching, skill-based coaching, medication, exercise.	Hypertension/T2DM	NA	None	Mobile application fostering self-management improved self-management behaviours HbA1c, HDL, LDL, and BP. No effect on BMI.
Chu-Hong et al. [27]	China	2015	RCT	360	40–75; group 1 x̄ = 53.4 (SD = 7.9, group 2 x̄ = 55.9 (SD = 7.8), group 3 x̄ = 53.8 (SD = 9.5)	Group1: female 55.2%, group2: female 60.5%, group3: female 62.4%	Reading materials to learn knowledge on hypertension via text messages on blackboards and booklets; public didactic lecture on hypertension invitation on phone; interactive education workshop on hypertension knowledge.	Hypertension	NA	None	Health educational interventions can greatly improve clinical outcomes in hypertensive patients. New methods of education should always be embraced.
Ahrari et al. [28]	Iran	2021	RCT	100	Control group x̄ = 54.44, intervention x̄ = 52.86	100	Self-care education on diabetes.	T2DM	No comorbidities	Not reported	Self-care education intervention has a huge impact on diabetics. Patient can better control of their condition and achieve better outcomes when it comes to HbA1c and quality of life.
Abraham et al. [29]	India	2020	RCT	80	Experimental group x̄ = 50.4 (SD = 9.3), control group x̄ = 50.6 (SD = 8.3)	56.25	4 sessions: diabetes education, goal setting, prioritising, and promoting diabetes care, barrier identification and problem-solving skills, managing diabetes-related distress and routine advice on diet, exercise, and medication.	T2DM	Hypertension and dyslipidaemia	Healthcare providers and caregivers	Psychological intervention with a cognitive and behavioural component can greatly affect glycaemic control, lifestyle, and quality of life of diabetic patients.
Thanh et al. [30]	Vietnam	2021	RCT	364	62.2	54.9	3-mo patient education and self-management on diet, exercise, drug therapy, adherence and knowledge.	T2DM	43.4% had no comorbidities, 34.6% had arterial Hypertension, 32.9% had lipid disorders	NA	Community education improves Hb1Ac, SBP, FBG and diabetes knowledge. No effect on weight. People most likely to benefit were people who already had a certain level of knowledge of their disease, who had a shorter duration of illness, and a lower baseline HbA1c.
Pienaar et al. [31]	South Africa	2021	RCT	288	54–69	Experimental group females 84.4%, control group females 83.7%	Peer-support by community health workers working at the same area at the patient along with usual care. Community health workers provided care via home visits, listening to the patients concerns, solving problems and giving sessions about diabetes, healthy eating, physical activity, and diabetes complications.	T2DM	Some had cardiovascular diseases, asthma, or arthritis	Healthcare provider and caregiver	Peer support will benefit a lot of diabetic patients. It can foster better follow-up with the disease. It keeps patients up to date.
Adepu et al. [32]	India	2021	RCT	158	53.04	45%	Patient follow-up, education regarding disease, medication, diet, and lifestyle modification were provided at discharge.	Hypertension/T2DM	NA	Healthcare provider	Post-discharge counselling by pharmacists positively impacts health-related quality of life and glycaemic control. Insignificant results were obtained for blood pressure control.
Alaofe et al. [44]	Benin	2021	Qualitative	32 patients with T2DM, 28 health care professionals	52.2	37.50%	Meta Salud diabetes was implemented in Benin to discuss T2DM education. It included diet, exercise, sugar monitoring, complications reduction, coping with stress. Facilitators of focus group were secondary school teachers with bachelor’s degrees in education.	T2DM	NA	NA	Meta Salud Diabetes should have a meal menu that considers guides, access, and acceptance. Group support and enjoyable sports are beneficial. Family should be involved in blood monitoring. Clear guidance on medication. Stress on being open with family. Fatalistic beliefs can be countered by religious spirituality. Patients were urged to express emotions and communicate as participants were reluctant to express feelings. Gender roles are to be considered.
Pamungkas et al. [45]	Indonesia	2022	Qualitative	22 patients 14 health care professionals	45	Patients: 62.5%, caretakers 87.5%	Based on McMaster’s family functioning theory: 1) problem-solving, 2) communication, 3) affective response, 4) family role in promoting healthy behaviour, 5) family involvement in glycaemic control, 6) behaviour control to promote healthy behaviour.	T2DM	NA	NA	Intervention that considers the cultural context of Indonesian communities is required.
Saleh et al. [41]	Indonesia	2021	Cross-sectional	68	60	60.3	No intervention	T2DM	NA	NA	Self-care, self-efficacy, and health literacy had a significant correlation with glycaemic control in older people with T2DM.
Gusty et al. [42]	Indonesia	2022	Cross-sectional	383	60.68	66.3	No intervention	Hypertension	NA	None	Hypertension patients in Indonesia do not have good knowledge of their disease. Weight management was the only component of self-care that showed correlation with knowledge.
Sari et al. [46]	Indonesia	2022	Qualitative	47	50.5	89.40%	The use of traditional medicine, the performance of physical activity, diet management, the performance of foot care, and participation in cultural events such as community gatherings and weddings, with special attention being paid to how participants dealt with food served at such events.	T2DM	NA	Seven health care providers had been taking care of diabetic patients, consisting of two physicians and five nurses.	T2DM patients find diet management difficult and are influenced by their cultural beliefs and practices. Furthermore, results show that patients coping with stress are influenced by a blend of culture and religion.
Hussien et al. [43]	Ethiopia	2021	Qualitative	11	56.7	63.63	Patient experiences with antihypertensive medication adherence, physical activity, diet adjustments, blood pressure monitors, adherence to prescribed medications, lifestyle modification, and self-monitoring practice of their blood pressure.	Hypertension	NA	NA	Facility-level problems are mainly responsible for poor self-management practice. This includes a shortage of medicines, high cost of medicines, and busyness of doctors due to high patient load, lack of appropriate education and counselling services, poor patient-provider interaction, and long waiting times. Future research and planning should focus on providing appropriate training for health care providers to enhance the patient-provider relationship and improve the supply of hypertensive medications.
Pamungkas et al. [47]	Indonesia	2019	Qualitative	8	45	Participants females 62.5%, family members females 87.5%	Application of a family function concept to help patients improve their health outcomes.	T2DM	NA	Healthcare provider	Some of the emerging themes included a lack of problem-solving skills for dealing with poor diabetes management, ineffective communication, and refusal to share the burden of diabetes management, lack of affective responsiveness in encouraging patients’ compliance, lack of affective involvement in diabetes management, insufficient family roles in supporting patients, poor behaviour control and insufficient family.
Tusubira et al. [48]	Uganda	2021	Qualitative	19	55	47	Social support for self-care.	Hypertension/T2DM	NA	Healthcare providers and caregivers	Patients preferred prescribed medicines as their first resort; traditional medicines were used in the case of the need for, as judged by the patient, a substitute/supplementation. Patients relied on social support from family members and patient peers to mitigate the impact of uncertain access to prescribed medicines.
Omidi et al. [33]	Iran	2018	RCT	80	60	78	Instructions on tensile training, strength, and exercise in the water. Hydrotherapy strengthens the muscles around the joint and reduces the pressure on it.	Knee osteoarthritis	NA	Healthcare provider	Health and medical personnel, including nurses, can take adequate measures to reduce patients' problems, particularly by using non-pharmacological methods such as self-management training.
Hendricks et al. [35]	South Africa	2022	RCT	100	50	100	Exercise, weight loss and the use of suitable footwear or bracing.	Knee osteoarthritis	50% had hypertension and 23% had diabetes mellitus type 2.	None	Education on non-pharmacological interventions improves pain and functionality outcomes in knee osteoarthritis patients.
Voloshyna et al. [34]	Ukraine	2018	RCT	88	43–75	60.23	An education project about a low-salt diet.	Hypertension	NA	NA	Low-salt diet education improves clinical hypertension outcomes. There is also an economic benefit as there will be diminishing rates of urgent cases and recurrent visits to the physician.
Khosravizade et al. [37]	Iran	2015	Quasi-experimental study	64	x̄ age experimental group = 51.2, x̄ age control group = 49.1	100	Education sessions were according to self-efficacy: practical achievement and improvement, verbal encouragement, and physical postures.	Hypertension	NA	NA	Hypertension education has a positive influence on systolic, diastolic, BMI, self-care, and self-efficacy behaviours.
Lamptey et al. [22]	LMICs	2022	Systematic review	1389	All included studies had a mean age of participants aged >45 y.	8 of the 9 included studies had a higher proportion of females.	Self-management education	T2DM	NA	Education sessions in various health care and non-health care settings.	There is an association between diabetes self-management education and glycaemic control.

BP – blood pressure, COPD – chronic obstructive pulmonary disease, FBG – fasting blood glucose, HbA1c – glycated haemoglobin, HDL – high-density lipoproteins, LDL – low-density lipoproteins, LMIC – low- and middle-income countries, NA – not available, RCT – randomised controlled trial, SBP – systolic blood pressure, SD – standard deviation, T2DM – type 2 diabetes mellitus, x̄ – mean

### Risk of bias (methodological quality of included studies)

**Figure 2:**
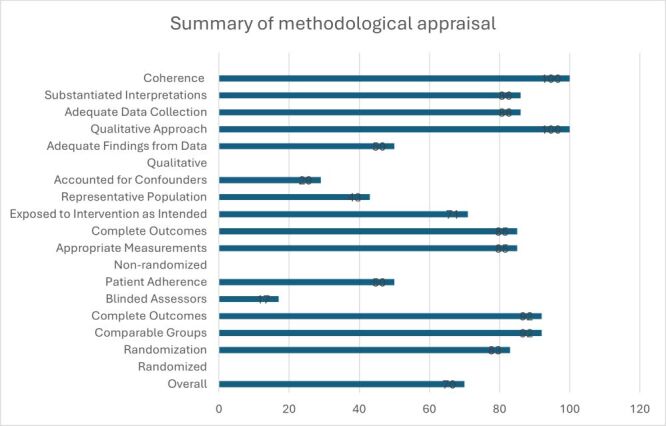
Risk of bias across included primary studies.

The results of the methodological quality appraisal of included studies are presented in the supplemental documents section (Appendix S2 in the **Online Supplementary Document**) and summarised for all included studies in **Figure 2**. Of the 12 included randomised trials, 83% performed randomisation using appropriate methods, 92% of the groups were comparable at baseline and reported complete outcomes. However, only 17% of them had outcome assessors blinded to the intervention, and only half (50%) of participants adhered to the assigned intervention. Furthermore, of the seven non-randomised quantitative studies that were included, 71% of these studies exposed their participants to the intervention as intended, but only 43% and 29% had participants that were representative of the population and accounted for confounders in their analysis, respectively. Most, 86–100% of the six qualitative studies were assessed as presenting an appropriate qualitative approach and adequate data collection methods. However, half of these (50%) failed to derive findings from collected data adequately. Data did not sufficiently substantiate interpretations.

### Interventions to facilitate self-management of chronic long-term conditions in LMICs

#### Characteristics of self-management interventions

Across studies and LTCS, three subtypes of self-management interventions were identified – self-management education, mobile Health (mHealth)-guided self-management, and community-based support for self-management. Self-management education and advice interventions covered topics such as diet, self-monitoring of disease outcomes, medication adherence, exercise, and a healthy lifestyle. Typically, they were delivered in the first instance by health care professionals (including pharmacists, family physicians, nurses, community health workers (CHWs), and cardiology residents) at different levels of training and qualifications across studies. There was no standardised delivery schedule for self-management education interventions across the different LTCS reported, such that frequency and duration of sessions varied significantly from ten minutes (individual one-on-one session only) to up to 30 minutes of group education, including up to four booster education sessions over 12 weeks.

mHealth-guided management and community-based support shared similar objectives with the educational interventions, with mHealth interventions utilising remote internet/telephone-based assistance, mobile applications and short message/messaging service (SMS) for monitoring and reminders, while CHWs supported patients in actual self-management activities in community-based initiatives, including home visits (Appendix S3 in the **Online Supplementary Document**).

### Effect of self-management interventions on health-related outcomes and quality of life

#### Diabetes

Overall, 12 studies (six trials [24–26,28,29,31], one quasi-experimental [36], and three cross-sectional [38,39,41]) explored the effectiveness of different self-management interventions among 2237 participants. Of the six randomised trials, four [24,28–30] reported on the effect of self-management education on glycaemic control (glycated haemoglobin (HbA1c) levels) of diabetic patients compared to usual care control). All the educational sessions in these trials were face-to-face sessions aimed at educating the patients on the cause/pathology of diabetes as an LTC, its complications, and both the pharmacological and lifestyle approaches to self-management. In a total of 672 diabetic patients, the trials reported statistically significant HbA1c levels reduction (*e.g.* from 9.45% to 8.98% HbA1c in Nazir et al. [24]; from 8.58% (standard deviation (SD) = 0.84) to 8.08% (SD = 0.81) HbA1c in Abraham et al. [29]) compared to 336 patients who did not undertake self-management education on diabetes.

Three trials [24,28,29] reported on quality-of-life outcomes after self-management education interventions. Two of these [24,28] reported positive improvement (EuroQol Five Dimension (EQ-5D) score = 0.77–0.89 and EQ-5D score = 0.47 (SD = 0.36) to 0.61 (SD = 0.29), respectively). However, a third trial [29] showed no significant difference in health-related quality of life was observed compared to a control group using the diabetes quality of life measure.

Similar to the trials, the quasi-experimental study of 120 participants showed that diabetes self-management education reduced Hb1Ac levels from 8.76 to 8.05% [36]. The three cross-sectional studies [38,39,41] reported a significant correlation between education on self-care practice and improved glycaemic control (correlation coefficient (r) = −0.168) [38].

Only two RCTs [25,26] explored self-directed self-management programmes, which were delivered/supported via mHealth, via telephone or app-based in LMICs for diabetic patients. The two trials that supported self-management via mHealth have a positive effect on glycaemic control. For instance, in a trial investigating 60 participants [26], diabetes care mobile application in Indonesia fostered improved glycaemic control (HbA1c improved from baseline 8.043 to 6.440% compared to 8.553 to 8.240% in control participants who received usual diabetes care). Similarly, Mohamed et al. [25] highlighted the importance of close follow-up and continuous education for achieving significant reductions in HbA1c levels (1.88% reduction in HbA1c in intervention group *vs* 1.89% increase in usual care group, *P* ≤ 0.001), indicating that SMS messages received by experimental groups showed promising effectiveness in reinforcing knowledge/capability for self-management.

Only one study [31] explored the effectiveness of community-based support for self-management for patients with diabetes. The trial involving 282 participants with diabetes showed that community health worker support visits alongside usual diabetes care did not have a significantly more positive effect on glycaemic control compared to usual care only [31] (*i.e.* 0.4% median (MD) improvement (interquartile range (IQR) = −0.5, 1) HbA1c improvement in intervention vs 0.2% MD improvement (IQR = −0.2, 0.8) in control, (*P* = 0.87).

There was no standardised content or format for incorporating self-management education interventions in core care plans for diabetic patients across the included studies. Though varied in terms of content and mode of delivery, self-management education and advice show the most promising improvement in clinical outcomes and health-related quality of life among diabetic patients in LMICs. Community-based support for self-management and self-directed self-management programmes delivered/supported online, via telephone or app-based may be best used to optimise education as a first-line self-management intervention for diabetic patients in LMICS. Only one of the 10 studies on diabetes health outcomes had an overall high risk of bias, with these 10 studies reporting consistent results (**Table 2**). The overall strength of the evidence on the effect of self-management interventions on health-related outcomes and quality of life among diabetic patients in LMICs was moderate.

**Table 2:** Summary of findings

Items	Evidence-based studies (n) and relevant sample size	Effects	Quality of studies (risk of bias)	Inconsistency	Strength of evidence (modified grade rating)*
Diabetes	10 studies (6 trials [24–26, 28–32], 1 quasi-experimental [36], 3 cross-sectional [38,39,41]); n = 2237	9.45% to 8.98% HbA1c in Nazir et al. [24]; from x̄ = 8.58% (SD = 0.84) to x̄ = 8.08% (SD = 0.81) HbA1c in Abraham et al. [29]	7 studies had an overall low risk of bias. 1 study had an overall high risk of bias	Consistently, studies reported an improvement in diabetes health outcomes through self-management.	Moderate
Hypertension	3 studies (2 trials [27,34], quasi-experimental [37]); n = 523	143.9 mm Hg to 134.8 mm Hg in Chu-Hong et al. [27]	1 study had overall low risk of bias, 2 had overall high risk of bias	Consistently, studies reported an improvement in hypertension health outcomes through self-management.	Low
Co-morbid long-term conditions	3 studies (2 trials [30,32], 1 cross-sectional [40]); n = 1091	8.21% to 7.67% HbA1c with 3.7 mm Hg reduction in blood pressure in Thanh et al. [30]. Adepu et al. [32] reported no significant improvement in blood pressure	1 study had overall low risk of bias, 2 studies had overall high risk of bias	The outcomes were inconsistent throughout the included studies on co-morbid long-term conditions.	Very low
Musculoskeletal pain health	2 studies (2 trials [33,35]); n = 180	There was a x̄ = 3.97 (SD = 0.91) to x̄ = 2.77 (SD = 1.03) pain intensity scores improvement after a self-management intervention in Omidi et al. [33]. 4.25 (experimental) *vs* 5.9 (control) pain severity scores in Hendricks et. al [35]	2 studies had overall low risk of bias	Consistently, studies reported an improvement in musculoskeletal pain health outcomes through self-management.	Low
Self-management barriers	12 studies (5 qualitative studies [43–47], 2 trials [25,32], 4 cross-sectional [38–40,42], 1 quasi-experimental study [36]); n = 2523	NA	3 studies had overall high risk of bias, 7 studies had overall low risk of bias	NA	Moderate
Self-management facilitators	7 studies (2 qualitative [44,48], 1 trial [27], 4 cross-sectional [38,40–42]); n = 2283	NA	1 study had overall high risk of bias3, 5 studies had overall low risk of bias	NA	Low
Cost feasibility	4 studies (2 qualitative [44,48], 2 RCTs [25,35]); n = 279	1581 Egyptian pound (about USD 100 according to the average exchange rate in the year 2021) reduction in cost per 1% HbA1c improved in Mohamed et al. [25]	4 studies had overall low risk of bias	NA	Low

HbA1c – glycated haemoglobin, NA – not applicable, RCT – randomised controlled trial, SD – standard deviation, x̄ – mean

*Conceptualisation – quality of evidence across studies: high – further research is very unlikely to change our confidence in the estimate of effect, moderate – further research is likely to have an important impact on our confidence in the estimate of effect and may change the estimate, low – further research is very likely to have an important impact on our confidence in the estimate of effect and is likely to change the estimate, very low – any estimate of effect is very uncertain.

#### Hypertension

The effectiveness of self-management interventions in improving clinical outcomes (blood pressure measured in millimetres of mercury (mmHg)) of patients living with hypertension in LMICs was explored by three of the included studies (two RCTs [27,34] and one quasi-experimental study [37]) involving a total of 523 participants. All the interventions across the three studies belonged to self-management education and advice subtypes. The two trials reported positive improvements in blood pressure for patients who had self-management education in addition to usual care. Specifically, the three-arm trial by Chu-Hong et al. [27] involved 360 patients with hypertension (group one utilised text messages that directed patients to text-based material, group two adapted a lecture-style education session (in groups), while group three participated in an interactive self-management education workshop. Group two and three patients showed significant improvements in blood pressure (143.9 to 134.8 mm Hg and 148.7 to 133.7 mm Hg of systolic blood pressure, respectively). Group one patients made no statistically significant improvements in their systolic blood pressure. The second RCT by Voloshyna et al. [34] involved 88 patients with poor blood pressure control at baseline in two experimental arms. One arm received general nutritional advice, which led to a fall below 140/90 mm Hg in 26% of the patients. On the other hand, group two patients received more focused nutritional education and advice on a low-sodium diet, leading to a fall below 140/90 mm Hg (*P* < 0.0001) for 64% of the patients.

The quasi-experimental study by Khosravizade et al. [37] investigated 32 hypertensive women and 32 usual care controls and found self-care education fostered statistically significant improvement in blood pressure, 129.7/83.7 mm Hg to 117.9/75.3 mm Hg.

Self-management education was found to be effective in improving blood pressure outcomes in hypertensive patients, with studies showcasing a preference for direct education sessions, including interactive styles over text messages (virtual assistance). Current evidence also shows that focused self-management education sessions may be more effective than multi-content general education sessions. The certainty/strength of the evidence was deemed low due to two studies having high risk of bias, although the results were consistent across the three studies contributing to the evidence base on the effect of self-management interventions for improving clinical outcomes (blood pressure) of patients living with hypertension in LMICs.

#### Comorbid LTCs

Three studies [30,32,40] investigated effects of self-management interventions involving participants with multiple /or co-morbid LTCs. Of these, two involved patients with diabetes and hypertension [30,32]. Employing a RCT study design, Thanh et al. [30] explored the effect of a self-management education programme on Hb1Ac levels of 364 diabetes patients, 34.6% of whom had co-morbid arterial hypertension. At the end of a three-month follow-up period, authors report improved Hb1Ac levels by 0.54% from a baseline HbA1c value of 8.21% and reduced systolic blood pressure by 3.7 mm Hg. However, Adepu et al. [32] found no significant difference in average blood pressure improvements for patients with hypertension (134.07/83.4 mm Hg to 126.37/78.59 mm Hg *vs* control 143.5/83.75 mm Hg to 143.21/83.73 mm Hg), and those with both hypertension and diabetes (132.78/82.5 mm Hg to 130.14/80 mm Hg *vs* control 138.8/84 mm Hg to 135.9/82.43 mm Hg) whilst exploring post-discharge counselling and education by pharmacists. On the other hand, participants with diabetes had improved glycaemic control (165.88 mgmes per decilitre (mg/dL) to 146.6 mg/dL capillary blood glucose) compared to control patients who only received medical advice on their last visit to the doctor. It is, however, important to consider the cofounding effect of differences in both inter-professional delivery and content of self-management education intervention in this trial.

Adepu et al. [32] also reported on the effects of post-discharge counselling and education by pharmacists (compared to usual care from doctors), on quality of life. The psychological general well-being index scores of patients living with hypertension (score 64.22 to score 79.25), and patients with diabetes and hypertension (score 17.47 to score 19.95) increased post-intervention, with patients living with diabetes (score 18.01 to score 20.35) also showing increased average scores in health-related quality of life (QoL).

In addition, the cross-sectional study by Qu et al. [40] involved 873 patients with hypertension, 54.1% of whom had co-morbidities (10.4% diabetes, 7.0% arthritis). The study showed that self-management education on medication management, diet, exercise, lifestyle, and risk factor management reduced the odds of having poor blood pressure control (odds ratio (OR) = 0.98), with poor control being defined as blood pressure >140/90 mm Hg.

The presence of co-morbidity or having multiple LTCs appears to moderate the effectiveness of self-management education and advice for improving clinical outcomes. However, available evidence shows that self-management education also leads to improved health-related quality of life of diabetics with co-morbid hypertension. There was no evidence regarding mHealth or community-based self-management support for co-morbid /multiple LTCs. The studies on co-morbid LTCs were inconsistent and two of the included three studies had a high risk of bias (very low strength of evidence).

#### Musculoskeletal pain conditions

Self-management education for improving health outcomes among people with musculoskeletal pain conditions was studied in 180 patients across two trials [33,35]. Specifically, a positive reduction in pain was reported among 80 knee osteoarthritis patients enrolled in a study to determine the effect of self-management training (diet, hydrotherapy, and exercise) on pain intensity. Mean (x̄) pain intensity scores decreased from x̄ = 3.97 (SD = 0.91) to x̄ = 2.77 (SD = 1.03) after the intervention in the experimental group, while x̄ pain scores did improve within the control group, but these were not significant [33]. Similarly, in a study investigating 100 women with chronic joint pain, 43.4% of which had osteoarthritis, advocating self-management (exercise, weight loss and suitable footwear/bracing) resulted in significant improvement in the primary outcome of function and disability (experimental group WHO Disability Assessment Score 2.0 score MD = 6 *vs* the conventional care group’s MD = 14), and pain severity (experimental group pain severity score score x̄ = 4.25 *vs* the conventional care group’s score x̄ = 5.9) [35]. Only one study, Hendricks et al. [35], explored the effect of osteoarthritis self-management on quality-of-life outcomes, reporting an improvement in EQ-5D scores (0.19–0.72 to a score of 0.58–0.85 post-intervention follow-up).

Self-management education has a positive effect on pain, functional and quality of life outcomes in patients living with osteoarthritis in LMICs. The strength of the evidence on the effect of self-management interventions for reducing pain and improving function for people living with osteoarthritis and joint pain in LMICs is moderate due to consistent results and low risk of bias.

### Barriers and facilitators of effective self-management among patients living with chronic LTCs in LMICs

#### Barriers

Across a total of 12 studies involving 2523 participants (five qualitative studies [43–47], two RCTs [25,32], four cross-sectional [38–40,42], and one quasi-experimental study [36]) reported on barriers to effective self-management programmes among patients living with chronic LTCs in LMICs. These barriers were broadly defined as lifestyle/individual, economic, social factors, and LTC condition-related (**Table 3**).

**Table 3:** Barriers to self-management

Category	Evidence	Socioeconomic details and comments on scalability
Lifestyle/individual	Forgetting about medication, lack of knowledge of foot care, no time for exercising, complexity of insulin and fear of hypoglycaemia were some of the reasons for patients not complying with self-management practice [39]. Univariate analysis in a randomised controlled trial showed that one of the most significant factors affecting HbA1C levels was eating habits [25]. With regards to components of self-management, a high percentage of participants failed to engage with: treatment management (59.54%), exercise (60.80%), lifestyle management (63.47%), diet (70.18%). Other least practised self-management components in another study of hypertension patients were medication compliance (46%), diet compliance (11.5%) and physical activity (13.1%) [42]. Another qualitative study utilised focus groups with diabetes patients, caretakers, and health care workers to investigate barriers. Highlights were a lack of problem-solving skills in disease management and poor behaviour control of type 2 diabetes mellitus [45]. From Sari et al. [46] qualitative study on diabetics emerged four themes of barriers to self-management, one of which was misconceptions about diabetes and medication management.	Study conducted in Pakistan: self-management programmes in Pakistan could capitalise on communal living styles by including community education interventions. Study conducted in Egypt: 40% illiterate. 48% unemployed. A pictorial diabetes care model was well received by Egyptian society. Study conducted in Indonesia: 88% have low education. 87% do not work. The involvement of patients and their families is needed to improve self-care behaviour. Study conducted in Indonesia: participants were patients, caretakers, and health care professionals. Family function influences the adoption and maintenance of healthy behaviours. Study conducted in Indonesia: 79% married. 60% of the education is below secondary level. 45% of housewives. Javanese culture (*e.g.* religion as a coping mechanism, cold rice does not increase blood glucose) had a considerable effect on the patients’ health status.
Economical	Insulin prescription access was one of the reasons for patients not complying with self-management practice [39]. Rural health insurance with limited outpatient benefits was associated with poor blood pressure control [40]. A qualitative study of focus groups on diabetes patients reported that the financial burden of healthy foods, medical equipment and treatment and distance from market (access and affordability issues) were barriers to self-management [44]. Healthcare providers reported a lack of manpower, time and knowledge [45]. Facility /infrastructure - associated barriers, as reported by participants (hypertensive patients in Ethiopia) [43] include shortage of medicines, high cost of medicines, busyness of doctors due to high patient load, lack of appropriate education and counselling services, poor patient-provider interaction, and long waiting times.	Study conducted in Pakistan: self-management programmes in Pakistan could capitalise on communal living styles by including community education interventions. Study conducted in China: 21% lower than high school. 17% employed. 68% retired. 35% earned less than USD 225. An approach that considers condition-specific factors, social/physical context, knowledge, beliefs, skills and target outcomes is effective in Chinese culture. Study conducted in Benin: 75% married. 16% retired. 44% employed. There is a need for a culturally sensitive and motivational (spiritual and emotional) approach in Benin. Study conducted in Indonesia: participants were patients, caretakers, and HCP. Family function influences the adoption and maintenance of healthy behaviours in Indonesia. Study conducted in Ethiopia: 45% no formal education. 73% urban living. Facility level barriers were identified as a major culprit for poor self-management practice.
Social	Themes related to social barriers include the complexity of information on treatment and disease management not fully addressed by doctors, social stigma of disease, and gender roles being an important consideration (*e.g*. women taking care of what the family eat) [44]. Ineffective communication and lack of effective family support [45]. Cultural beliefs and practices not aligned with prescribed treatment regimen (*e.g.* use of medicinal plants to lower blood pressure), coping influenced by a blend of culture and religion and cultural influence on diet management [44] and possible social exclusion [47].	Study conducted in Benin: 75% married. 16% retired. 44% employed. There is a need for a culturally sensitive and motivational (spiritual and emotional) approach in Benin. Study conducted in Indonesia: participants were patients, caretakers, and HCP. Family function influences the adoption and maintenance of healthy behaviours in Indonesia. Study conducted in Indonesia. Participants were patients, caretakers, health volunteers, and HCPs. Partly due to cultural and historic perceptions of ‘doctors know best’ and paternalism, self-management is an emerging concept in health care settings. Research into patient empowerment and how to engage with health professionals could be helpful.
Condition related	Psychological distress and decreased glycaemic control may moderate commitment to self-management interventions [38]. Participants undergoing treatment for comorbid diseases (*vs* those who were not) had significantly higher HbA1c values [36]. Longer disease duration was associated with poor blood pressure control [40]; by implication, patients may be discouraged and lose motivation for self-management over time. Adepu et al. [32] trial showed that co-morbid hypertension has a negative effect on diabetic clinical outcomes of patients managed via self-management. In addition, there is a lack of knowledge on the severity of diabetes (/or prognosis), and the skill to carry out self-management practices [47].	Study conducted in Jordan: 51% below secondary level education. Mean USD 439 moly household income. 68% unemployed. Culturally sensitive programmes delivered by qualified diabetes educators are appropriate in Jordan. Study conducted in Egypt: 57% lower than secondary level education. 55% used free governmental health sources. Study conducted in China: 21% lower than high school. 16.6% employed. 68% retired. 35% earned less than USD 225. An approach that considers condition-specific factors, social/physical context, knowledge, beliefs, skills and target outcomes is effective in Chinese culture. Study conducted in India: 61% under secondary school education. 18% employed. 37% of housewives. Study conducted in Indonesia: participants were patients, caretakers, health volunteers, and HCPs. Refer to the last row in the social section of this table.

HbA1c – glycated haemoglobin, HCP – health care professional

#### Facilitators

Of included studies, seven in a total of 2283 patients (two qualitative [44,48], one RCT [27], and four cross-sectional [38,40–42]) explored facilitating factors for self-management intervention practices among patients living with LTCs in LMICs. For example, Al Momani et al. [38] found that higher income, higher education, employment, lower body mass index (BMI), and oral hypoglycaemic agents treatment without insulin were associated with lower HbA1c levels in Jordanian society. Being females were associated with higher self-management engagement scores [40]; this was true for a majority of the retired and above high school educated study sample. Furthermore, groups (majority with education below high school level) with more interactive activities (listening to someone speaking, taking part in the workshop) had better clinical outcomes, rather than didactic learning (reading booklets and messages) [27]. Similarly, Alaofe et al. [44] analysis, who had only 44% employed and 16% retired participants, identified community support, easier and enjoyable physical activities as facilitators to physical activity. The availability and accessibility of medical equipment fostered better self-management practices at home for some patients.

Religion seemed to be the most common coping mechanism, followed by focusing on what is controllable. Saleh et al. [41] found that self-efficacy and health literacy were positively correlated with normal HbA1c levels in an Indonesian sample where 46% of participants were unemployed. Gusty et al. [42] reported that in hypertension patients (who had low education and were mostly unemployed), there is a relationship between knowledge and adherence to weight management, with other components of self-care not showing any significant correlation with knowledge. Finally, Tusubira et al. [48] conducted a qualitative analysis in Uganda, where 53% of participants had below secondary level education and 53% were farmers. They found that patients preferred prescribed medicines as their first resort, but also used traditional medicines to get around the inconsistency of access to prescribed medicines or as a supplement to those medicines. They also relied on social support to mitigate the impact of inconsistent access to prescribed medicines. This could also be a barrier as patients may share prescription medicines.

### The feasibility (cost to individuals, cost of interventions/health care service) of self-management interventions for chronic LTCs in LMICs

Outcomes of self-management in terms of economic feasibility were reported in four of the included studies (two qualitative studies [44,48], and 2 RCTs [25,35]). Alaofe et al. [44] found that financial burdens associated with the cost of healthy foods and equipment were significant barriers to adhering to self-management education and advice. Tusubira et al. [48] reported that ‘vernacular practices’ (*i.e.* local/traditional medicines or practices) were often used to supplement treatment when basic equipment or materials were unavailable. Hendricks [35] identified travel costs to and from community health centres (where self-management requires access to facilities) as a significant barrier for women with chronic joint pain/arthritis. Specifically, Mohamed et al. [25] conducted an incremental cost-effectiveness analysis and found that the use of comprehensive patient-centred care (face-to-face individual patient education) significantly reduced the average cost of medicines per patient, frequency of dosage, and insulin doses monthly, with a reduction of 1581 Egyptian pound per 1% HbA1c reduction for patients maintained on the programme for three months compared to usual care. Self-management is evidenced as a useful tool in reducing personal costs and out-of-pocket expenses relating to living with LTCs in LMIC settings.

## DISCUSSION

In this study, we have systematically identified, appraised, and synthesised current evidence regarding outcomes of self-management intervention among patients living with LTCs in LMIC settings. The barriers and facilitators of successful self-management and the feasibility of these interventions were also described. Based on available evidence, profiled self-management interventions led to modest but improved clinical outcomes and quality of life for patients with diabetes, hypertension, and osteoarthritis. These results are consistent with Lamptey et al.’s [22] scoping review. Although there was significant heterogeneity in reporting of the demography of study participants, most studies included patients from a wide array of backgrounds (socio-economic, education, and marital status), and gender. Our findings are therefore applicable to most people in LMICs context though methodological limitations of included primary studies forming the evidence base implies findings must be interpreted with caution.

As indicated in the evidence base, self-management for diabetes was most prevalent. On one hand this shows increasing awareness of diabetes as a common LTC in LMICs but also shows a huge gap in practice – a lack of standardised approach and incorporation of self-management education as a core treatment for all patients living with diabetes in LMICs. Living with one or multiple LTCs is difficult, affecting almost every facet of life for patients and their careers. It is also a long-term sail meaning that supporting people with LTCs on a journey of self-activation will help them feel more in control and live a better life. Though studies reported on disease specific clinical outcomes and quality of life, follow-up reports on these outcomes were non-existent or were of short duration (maximum 12 weeks). Since none of the studies explicitly investigated nor reported on patient activation and readiness to engage in self-management in the longer term, the successes reported in these studies will therefore need to be interpreted with caution.

In the context of self-management, it is essential to consider both barriers and facilitators of self-care. Despite this review found no studies reporting on self-management of mental health problems as a LTC in LMICs, psychological distress was reported as a major barrier to self-management, but no study reported patients with reported LTCs (*i.e.* diabetes, hypertension, arthritis) having been diagnosed with mental health problems such as anxiety and depression. Given current evidence on the existence (co-morbidity) of anxiety and depression with physical LTCs such as diabetes, hypertension, and musculoskeletal pain in HICs [18–20], it is highly likely that these mental health problems are being missed among people with LTCs in LMICs. Systematic omission of these evidently compromises vital aspects of person-centred care. Lack of education /awareness among health professionals and development of self-management strategies that incorporate mental and physical health may negatively impact on the health of people living with LTCs and further increase the overall burden in LMICs.

Overall, the evidence on economic feasibility was scarce. Out of the 26 studies included in our analysis, only one study evaluated the decrease in cost associated with patient self-management interventions. The intervention resulted in reduced medication usage in the future, which was a contributing factor to the financial impact of living with LTCs in a low-resource setting. The paucity of reports with standardised cost-effectiveness units (*e.g.* cost per unit of Hba1c improvement) hindered attempts at comparison across different self-management interventions. Careful investments in self-management interventions could potentially reduce further unnecessary health care utilisation in already over-stretched health systems.

### Strengths and limitations

This review provides a summary of the current evidence on the effectiveness, facilitators, and barriers of self-management of LTCs in LMICs, drawing together, for the first time, findings from the most common LTCs *i.e.* diabetes, hypertension, and arthritis. Other strengths of this review include a comprehensive search of the literature, an evidence synthesis incorporating all available evidence (experimental and qualitative studies) and conducted in line with robust systematic review methodology.

However, there are also limitations to our study. Large heterogeneity in study design and reporting from primary studies precluded intended statistical pooling meta-analysis and grading of evidence. There are currently no core outcomes set for evaluating self-management programmes with cultural sensitivities in LMICs. This is needed to facilitate robust methodological evaluation and inform policy and clinical decision-making. Future research should also investigate the cost-effectiveness of self-management interventions using quantitative and standard units like cost per percentage improvement in biological markers (*e.g.* mmHg) or dollars per life-year gained.

The minimal age of 45 years in our eligibility criteria also implied that some evidence of self-management interventions in LMICs among younger patients was not represented in this review. Given factors such as literacy, self-efficacy, motivation and increasing use of mobile technology in LMICs, approaches to self-management interventions which will be readily feasible, successful and scalable among younger populations with LTCs are likely to be different to those of older populations. This is an important focus for future primary research and systematic review of evidence from LMICs.

## CONCLUSIONS

Self-management education and advice shows promise for improving outcomes for people with common LTCs in LMICs. mHealth-guided self-management and community-based support are best used as a complementary approach to optimising care. Beyond diabetes and hypertension, further intervention development and implementation studies on self-management in LMICs (including mental health and musculoskeletal health self-management protocols) are warranted but there needs to be first an agreement and specification of core-outcomes set for evaluating self-management interventions among people living with LTCs in LMICs. There is a need for regulatory bodies to develop clinical guidelines and promote implementation of tailored self-management education as a core management strategy tailored for LTCs care in LMICs.

Support initiatives to improve access to reliable health care information through education and advice should be seen as a basic fundamental right of every patient. This is especially true for patients living with LTCs in LMICs. This will combat myths and misinformation, uninformed practices that could further lead to deterioration/poorer health and quality of life outcomes. Ensuring that health workers have access to reliable health care information and have sufficient time in consultations to do so should be a moral obligation of government and regulatory bodies involved in provision of health care services. For instance, there is currently no clinical practice guideline on the management of multiple LTCs in LMICs. Given that most LTCs share similar risk factors, adopting a person-cantered care approach with conscientiously developed self-management education and advise as a core management is a first step. This will need to be closely followed by proper implementation plan to deliver this at the grassroots level across health systems parastatals in LMICs. One of such infrastructures that could be employed is community-based LTC management centres to empower patients with knowledge for improving their health and QoL outcomes. Here, culturally appropriate practical workshops and creative learning can easily be implemented to promote knowledge and patient activation for self-management.

## Additional material


Online Supplementary Document
