# Supplementary material for: Self-management interventions for common long-term conditions in low- and middle-income countries: a synthesis of current evidence
Source: J Glob Health. 2025 May 23;15:04148. doi: 10.7189/jogh.15.04148 (PMC12099277; doi:10.7189/jogh.15.04148)
Supplement: Online Supplementary Document [file jogh-15-04148-s001.zip › jogh-15-04148-s001.pdf]

Supplement to: Akhal T, Gabra M, Adesokan M, Babatunde OO. Self-management interventions for common long-term conditions in low- and middle-income countries: a synthesis of current evidence. J Glob Health. 2025;15:04148.

Appendix S1. Detailed MEDLINE Search Strategy

Ovid MEDLINE(R) ALL <1946 to July 01, 2022>

1 Self Care/ 35406

2 Self-Management/ 4634

3 Self Medication/ 4933

4 ("self care" or "self management" or "self medication" or "self car$" or "self help" or "self guided" or "self directed" or "self manag$").ab,kf,ti. 63081

5 ((patient$ adj3 directed) or (patient adj3 guided)).mp. or (pyschoeducation$ or psycho educatin$).ab,kf,ti. [mp=title, abstract, original title, name of substance word, subject heading word, floating sub-heading word, keyword heading word, organism supplementary concept word, protocol supplementary concept word, rare disease supplementary concept word, unique identifier, synonyms] 4604

6 ("self improvement" or (patient$ adj3 educat$) or (patient adj3 teach$) or (patient adj3 train$)).ab,kf,ti. 53254

7 ("expert patient$" or "lay led" or "peer led").ab,kf,ti. 1684

8 (patient$ adj3 (focus$ or participat$ or centr$ or centr$ or empower$ or support$ or collaborat$ or co-operat$ or cooperat$)).ab,kf,ti. 141581

9 1 or 2 or 3 or 4 or 5 or 6 or 7 or 8 269856

10 Back Pain/ 18547

11 ((dorsalgia or (backache or back ache) or (back or lumb$)) adj3 pain).ab,kf,ti. 61882

12 (((Spine or spinal) adj3 pain) or Coccyx or coccydyina).ab,kf,ti. 8291

13 Sciatic Neuropathy/ 2190

14 (sciatica or spondylosis or lumbago or "back disorder$").ab,kf,ti. 10927

15 Intervertebral Disc Displacement/ 19770

16 ((disk$ or disc$) adj3 (hernia$ or prolapse$ or slipped)).ab,kf,ti. 15514

17 Radiculopathy/ 5645

18 radicul$.ab,kf,ti. 17249

19 Osteoarthritis/ 41523

20 (Osteoarthr$ or "O.A" or arthrosis).ab,kf,ti. 106545

21 (degenerative adj (arthritis or joint or joints)).ab,kf,ti. 4959

22 Knee/ or Knee Joint/ 73545

23 Hip Joint/ or Hip/ 40090

24 (Knee$1 or hip$1).ab,kf,ti. 309034

25 Osteoarthritis, Knee/ 25024

26 Osteoarthritis, Hip/ 9449

27 ((knees$1 or hips$1) adj5 (pain or painful)).ab,kf,ti. 1519

28 "Hip and knee OA".ab,kf,ti. 423

29 (back pain or hip or knee OA).ab,kf,ti. 219114

30 Comorbidity/ 122135

31 (comorbid$ or morbid$).mp. or (multimorbid$ or multi morbid$).ab,kf,ti. [mp=title, abstract, original title, name of substance word, subject heading word, floating sub-heading word, keyword heading word, organism supplementary concept word, protocol supplementary concept word, rare disease supplementary concept word, unique identifier, synonyms] 756059

32 (multiple adj2 (disease$ or condition$ or illness$ or diagnosis$)).ab,kf,ti. 31800

33 (multipatholog$ or multi patholog$).ab,kf,ti. 61

34 Diabetes Mellitus/ 131467

35 Diabetes Insipidus/ 6393

36 (Diabet$ or prediabet$ or (DMi or " DM I")).ab,kf,ti. 736354

37 ("DM1or DM 1" or (DMii or DM ii)).mp. or (DM2 or "DM2").ab,kf,ti. [mp=title, abstract, original title, name of substance word, subject heading word, floating sub-heading word, keyword heading word, organism supplementary concept word, protocol supplementary concept word, rare disease supplementary concept word, unique identifier, synonyms] 2579

38 (DM adj2 type$).ab,kf,ti. 5875

39 Hyperglycemia/ 30703

40 (hyperglyc?emi$ or hyper glyc?emi$).ab,kf,ti. 70989

41 ((high$ or elevate$ or raise$) adj blood adj (fat$ or lipid$)).ab,kf,ti. 258

42 Overweight/ 30477

43 (Obes$ or (overweight or over weight) or (weight gain or weight loss) or fatness).ab,kf,ti. 494247

44 Hyperlipidemias/ 28145

45 Dyslipidemias/ 13692

46 (Hyperlipid?emi$ or hyper lipid?emi$).ab,kf,ti. 35105

47 ((high$ or elevate$ or raise$) adj blood adj (fat$ or lipid$)).ab,kf,ti. 258

48 Cardiovascular Diseases/ 168348

49 Cardiovascular.ab,kf,ti. 523629

50 ((heart or cardiac) adj3 failure).ab,kf,ti. 216297

51 (Hypertens$ or ((high$ or elevate$ or raise$) adj blood pressure)).ab,kf,ti. 500486

52 (myocardi$ adj3 infarction).ab,kf,ti. 202324

53 ("heart attack" or angina).ab,kf,ti. 61425

54 ((heart or coronary) adj3 disease).ab,kf,ti. 294433

55 Angina, Stable/ or angina.mp. 73172

56 Depression/ 142040

57 Depressive Disorder/ 74713

58 (depressed or depression or depressive).ab,kf,ti. 503475

59 Anxiety/ or Anxiety Disorders/ 130298

60 (Anxiety or (anxiet$ or anxious$)).ab,kf,ti. 248204

61 10 or 11 or 12 or 13 or 14 or 15 or 16 or 17 or 18 or 19 or 20 or 21 or 22 or 23 or 24 or 25 or 26 or 27 or 28 or 29 or 30 or 31 or 32 or 33 or 34 or 35 or 36 or 37 or 38 or 39 or 40 or 41 or 42 or 43 or 44 or 45 or 46 or 47 or 48 or 49 or 50 or 51 or 52 or 53 or 54 or 55 or 56 or 57 or 58 or 59 or 60 3986778

62 Developing Countries/ 79564

63 (middle income$ adj (countr$ or nation or nations or econom$)).ab,kf,ti. 29129

64 (developing adj (countr$ or nation or nations or econom$)).ab,kf,ti. 100513

65 ((under developed or underdeveloped) adj (countr$ or nation or nations or econom$)).ab,kf,ti. 1401

66 (3rd world adj (countr$ or nation or nations or econom$)).ab,kf,ti. 60

67 (third world adj (countr$ or nation or nations or econom$)).ab,kf,ti. 1167

68 (LAMI adj (countr$ or nation or nations or econom$)).ab,kf,ti. 53

69 (LMIC adj (countr$ or nation or nations or econom$)).ab,kf,ti. 88

70 (poor income adj (countr$ or nation or nations or econom$)).ab,kf,ti. 5

71 (low$ income adj (countr$ or nation or nations or econom$)).ab,kf,ti. 9550

72 (less$ developed adj (countr$ or nation or nations or econom$)).ab,kf,ti. 1592

73 (least developed adj (countr$ or nation or nations or econom$)).ab,kf,ti. 315

74 Asia, Southeastern/ or Asia, Northern/ or Asia/ or Asia, Western/ or Asia, Central/ 38278

75 Africa, Eastern/ or Africa, Southern/ or Africa/ or "Africa South of the Sahara"/ or Africa, Central/ or Africa, Northern/ or Africa, Western/ 53989

76 Caribbean Region/ 5257

77 West Indies/ 3660

78 Central America/ 4065

79 Latin America/ 12622

80 South America/ 10668

81 Europe, Eastern/ 4492

82 ((South or Southern) adj Asia$).ab,kf,ti. 17643

83 ((South East$ or Southeast$) adj Asia$).ab,kf,ti. 24924

84 (West$ Asia$ or africa$).ab,kf,ti. 277765

85 ((Middle or Far) adj East$).ab,kf,ti. 21859

86 ((Latin or Central or South) adj America$).mp. or (Caribbean$ or West Indies).ab,kf,ti. [mp=title, abstract, original title, name of substance word, subject heading word, floating sub-heading word, keyword heading word, organism supplementary concept word, protocol supplementary concept word, rare disease supplementary concept word, unique identifier, synonyms] 82648

87 East$ Europe$.ab,kf,ti. 11194

88 (Afghan$ or Angola$ or Armenia$).mp. or (Bangladesh$ or Benin$ or Bhutan$).ab,kf,ti. [mp=title, abstract, original title, name of substance word, subject heading word, floating sub-heading word, keyword heading word, organism supplementary concept word, protocol supplementary concept word, rare disease supplementary concept word, unique identifier, synonyms] 38651

89 (Bolivia$ or Burkina Faso$ or Upper Volta$).mp. or (Burundi$ or Cabo Verd$ or Cape Verd$).af. [mp=title, abstract, original title, name of substance word, subject heading word, floating sub-heading word, keyword heading word, organism supplementary concept word, protocol supplementary concept word, rare disease supplementary concept word, unique identifier, synonyms] 12739

90 (Cambodia$ or Cameroon$ or Central African Republic$).af. 21896

91 (Chad$ or Comoros or Comorian or Congo$ or (Cote d'Ivoire or Ivory Coast or Ivorian$)).mp. or (Djibouti$ or Egypt$ or El Salvador$).af. [mp=title, abstract, original title, name of substance word,

subject heading word, floating sub-heading word, keyword heading word, organism supplementary concept word, protocol supplementary concept word, rare disease supplementary concept word, unique identifier, synonyms] 194263

92 (Eritrea$ or Ethiopia$ or Gambia$).mp. or (Georgia$ or Ghana$ or Guatemala$).af. [mp=title, abstract, original title, name of substance word, subject heading word, floating sub-heading word, keyword heading word, organism supplementary concept word, protocol supplementary concept word, rare disease supplementary concept word, unique identifier, synonyms] 249084

93 (Guinea$ or Guyan$ or Haiti$ or Hondura$).mp. or (India$ or Indonesia$ or Jordan$).af. [mp=title, abstract, original title, name of substance word, subject heading word, floating sub-heading word, keyword heading word, organism supplementary concept word, protocol supplementary concept word, rare disease supplementary concept word, unique identifier, synonyms] 1340484

94 (Kenya$ or Kiribati$ or Korea$).mp. or (Lesotho or Mosotho or Basotho).af. [mp=title, abstract, original title, name of substance word, subject heading word, floating sub-heading word, keyword heading word, organism supplementary concept word, protocol supplementary concept word, rare disease supplementary concept word, unique identifier, synonyms] 131939

95 (Kosov$ or Kyrgyz$ or Lao$).mp. or (Malawi$ or Mali or Mali's or Malian$ or Mauritania$).af. [mp=title, abstract, original title, name of substance word, subject heading word, floating sub-heading word, keyword heading word, organism supplementary concept word, protocol supplementary concept word, rare disease supplementary concept word, unique identifier, synonyms] 34522

96 (Micronesia$ or Moldov$ or Mongolia$).mp. or (Morocc$ or Mozambi$ or Myanmar$ or Burma$ or Burmese$).af. [mp=title, abstract, original title, name of substance word, subject heading word, floating sub-heading word, keyword heading word, organism supplementary concept word, protocol supplementary concept word, rare disease supplementary concept word, unique identifier, synonyms] 57759

97 (Nepal$ or Nicaragua$ or Niger$).mp. or (Pakistan$ or PNG$ or Paraguay$ or Philippines$ or Filipino$).af. [mp=title, abstract, original title, name of substance word, subject heading word, floating sub-heading word, keyword heading word, organism supplementary concept word, protocol supplementary concept word, rare disease supplementary concept word, unique identifier, synonyms] 209073

98 (Rwanda$ or Samoa$ or Sao Tome$ or Principe$).mp. or (Senegal$ or Sierra Leone$ or Solomon Island$).af. [mp=title, abstract, original title, name of substance word, subject heading word, floating sub-heading word, keyword heading word, organism supplementary concept word, protocol supplementary concept word, rare disease supplementary concept word, unique identifier, synonyms] 28439

99 (Somalia$ or Sudan$ or Sri Lanka$).mp. or (Swaziland$ or Syria$ or Tajik$).af. [mp=title, abstract, original title, name of substance word, subject heading word, floating sub-heading word, keyword heading word, organism supplementary concept word, protocol supplementary concept word, rare disease supplementary concept word, unique identifier, synonyms] 45482

100 (Tanzania$ or Timor$ or Togo$ or Tonga$).mp. or (Tunisia$ or Uganda$ or Ukrain$).af. [mp=title, abstract, original title, name of substance word, subject heading word, floating sub-heading word, keyword heading word, organism supplementary concept word, protocol supplementary concept word, rare disease supplementary concept word, unique identifier, synonyms] 195327

101 (Uzbek$ or Vanuatu$ or Vietnam$ or (West Bank$ or Gaza$ or Palestin$)).mp. or (Yemen$ or Zambia$ or Zimbabwe$).af. [mp=title, abstract, original title, name of substance word, subject heading word, floating sub-heading word, keyword heading word, organism supplementary concept word, protocol supplementary concept word, rare disease supplementary concept word, unique identifier, synonyms] 60064

102 62 or 63 or 64 or 65 or 66 or 67 or 68 or 69 or 70 or 71 or 72 or 73 or 74 or 75 or 76 or 77 or 78 or 79 or 80 or 81 or 82 or 83 or 84 or 85 or 86 or 87 or 88 or 89 or 90 or 91 or 92 or 93 or 94 or 95 or 96 or 97 or 98 or 99 or 100 or 101 2808055

103 9 and 61 and 102 8638

Supplement to: Akhal T, Gabra M, Adesokan M, Babatunde OO. Self-management interventions for common long-term conditions in low- and middle-income countries: a synthesis of current evidence. J Glob Health. 2025;15:04148.

Appendix S2. The quality of individual studies.

1st Author, Year	Study Design	**Screening** (for all study types)			**Quantitative randomised Controlled trials**							**Qualitative**													
**Study**		**Methodological Quality Appraisal MMAT tool**																							
		Are there clear research questions? (Yes/No)	Do the collected data allow to address the research questions? (Yes/No)	Optional comments	Is randomization appropriately performed?	Are the groups comparable at baseline?	Are there complete outcome data?	Are outcome assessors blinded to the intervention provided?	Did the participants adhere to the assigned intervention?	**Optional comments**	**Score %**	Is the qualitative approach appropriate to answer the research question?	Are the qualitative data collection methods adequate to address the research question?	Are the findings adequately derived from the data?	Is the interpretation of results sufficiently substantiated by data?	Is there coherence between qualitative data sources, collection, analysis and interpretation?	**Optional comments**	**Score %**	Are the participants representative of the target population?	Are measurements appropriate regarding both the outcome and intervention (or exposure)?	Are there complete outcome data?	Are the confounders accounted for in the design and analysis?	During the study period, is the intervention administered (or exposure occurred) as intended?	**Optional comments**	**Score %**
Almomani, 2021	Cross-sectional	Yes	Yes																Yes	Yes	Yes	No	Yes		80%
Rahmatullah, 2021	Cross-sectional	Yes	Yes																No	Yes	Yes	Yes	Yes		80%
Nazir, 2020	Randomized Controlled Trial	Yes	Yes		Yes	Yes	No	No	N/R		40%														
Farag Mohamed, 2021	Randomized Controlled Trial	Yes	Yes		Yes	Yes	Yes	No	Yes		80%														
Qu, 2019	Cross-sectional	Yes	Yes																Yes	No	Yes	Yes	Yes		80%
Emara, 2021	Quasi-experimental Study	Yes	Yes																No	Yes	Yes	No	Yes		60%
Pamungkas, 2022	Randomized Controlled Trial	Yes	Yes		Yes	No	Yes	N/R	N/R		40%														
Chu-Hong, 2015	Randomized Controlled Trial	Yes	Yes		Yes	Yes	Yes	No	Yes		80%														
Ahrari, 2021	Randomized Controlled Trial	Yes	Yes		Yes	Yes	Yes	No	Yes		80%														

1st Author, Year	Study Design	Screening (for all study types)			Quantitative randomised Controlled trials							Qualitative						
	**Study**																	N
		Are there clear research questions? (Yes/No)	Do the collected data allow to address the research questions? (Yes/No)	Optional comments	Is randomization appropriately performed?	Are the groups comparable at baseline?	Are there complete outcome data?	Are outcome assessors blinded to the intervention provided?	Did the participants adhere to the assigned intervention?	**Optional comments**	**Score %**	Is the qualitative approach appropriate to answer the research question?	Are the qualitative data collection methods adequate to address the research question?	Are the findings adequately derived from the data?	Is the interpretation of results sufficiently substantiated by data?	Is there coherence between qualitative data sources, collection, analysis and interpretation?	**Optional comments**	**Score %**
Hussein,2021	Qualitative	Yes	Yes									Yes	Yes	N/R	N/R	Yes		60%
Pamungkas, 2021	Qualitative	Yes	Yes									Yes	N/R	No	Yes	Yes		60%
Tusubira, 2021	Qualitative	Yes	Yes									Yes	Yes	Yes	Yes	Yes		100%
Omidi, 2018	Randomized Controlled Trial	Yes	Yes		Yes	Yes	Yes	Yes	N/R		80%							
Voloshyna, 2018	Randomized Controlled Trial	Yes	Yes		N/R	Yes	Yes	N/R	N/R		40%							
Hendricks, 2022	Randomized Controlled Trial	Yes	Yes		Yes	Yes	Yes	Yes	Yes		100%							

1st Author, Year	Study Design	**Screening** (for all study types)			**Quantitative randomised Controlled trials**							**Qualitative**													
**Study**		**Methodological Quality Appraisal MMAT tool**																							
		Are there clear research questions? (Yes/No)	Do the collected data allow to address the research questions? (Yes/No)	Optional comments	Is randomization appropriately performed?	Are the groups comparable at baseline?	Are there complete outcome data?	Are outcome assessors blinded to the intervention provided?	Did the participants adhere to the assigned intervention?	**Optional comments**	**Score %**	Is the qualitative approach appropriate to answer the research question?	Are the qualitative data collection methods adequate to address the research question?	Are the findings adequately derived from the data?	Is the interpretation of results sufficiently substantiated by data?	Is there coherence between qualitative data sources, collection, analysis and interpretation?	**Optional comments**	**Score %**	Are the participants representative of the target population?	Are measurements appropriate regarding both the outcome and intervention (or exposure)?	Are there complete outcome data?	Are the confounders accounted for in the design and analysis?	During the study period, is the intervention administered (or exposure occurred) as intended?	**Optional comments**	**Score %**
Abraham, 2020	Randomized Controlled Trial	Yes	Yes		Yes	Yes	Yes	No	Yes		80%														
Thanh, 2021	Randomized Controlled Trial	Yes	Yes		Yes	Yes	Yes	N/R	N/R		60%														
Pienaar M., 2021	Randomized Controlled Trial	Yes	Yes		Yes	Yes	Yes	No	Yes		80%														
Adepu, 2021	Randomized Controlled Trial	Yes	Yes		N/R	Yes	Yes	N/R	N/R		40%														
Alaofe, 2021	Qualitative	Yes	Yes									Yes	Yes	Yes	Yes	Yes		100%							
Pamungkas, 2022	Qualitative	Yes	Yes									Yes	Yes	No	Yes	Yes		80%							
Saleh,2021	Cross-sectional	Yes	Yes																Yes	Yes	Yes	No	N/R		60%
Gusty, 2022	Cross-sectional	Yes	Yes																No	Yes	Yes	No	Yes		60%
Khosravizade, 2015	Quasi-experimental Study	Yes	Yes																No	Yes	No	No	N/R		20%
Sari, 2022	Qualitative	Yes	Yes									Yes	Yes	Yes	Yes	Yes		100%							

Appendix S3. Self-management intervention characteristics

Disease	Type of intervention	Details of intervention		Delivered by	Outcomes assessment	Facilitators/Barriers	Reference
		mode	*duration*				
Diabetes	1st group: Education	Focus groups / Interviews / Articles / education applications / Online consultations / Posters about diabetes / Face-to-Face education / Post discharge counselling / Self-care training and education course.	2 weeks; 10 minutes a session (Nazir) / 12 weeks; a session a week (Emara) / 6 Sessions (Guevara) / 4 sessions (Abraham) / 3 months (Thanh) / 2 months (Adepu).	Pharmacists / Family physicians / Nurses / Secondary school teachers with bachelor's degrees.	Pharmacists / Observed by physicians / Consultation by a diabetes care professional.	*Facilitators:* Face-to-face training by pharmacists / /// *Barriers:* Some pharmacists believe such interventions are outside the scope of conventional pharmacists' practice / community pharmacists might not be well-suited in all countries / *When interventions were assessed/restricted to a specific hospital, the data could not be generalized to the lifestyle and knowledge of all locals.*	Almomani, 2021 / Nazir, 2020 / Emara, 2021 / Pamungkas, 2022 / Abraham, 2020 / Thanh, 2021 / Adepu, 2021 / Alaofe, 2021 / Pamungkas, 2022 / Ahrari, 2021
		Focus groups / Interviews / Articles / education applications / Online consultations / Posters / Advice.					Almomani, 2021 / Rahmatullah, 2021 / Pamungkas, 2022 / Abraham, 2020
		Advice about smoking, nutrition, diet, and lifestyle.				*Barriers:* seasonality and distance were barriers to obtaining healthy food.	Almomani, 2021 / Pamungkas, 2022 / Farag Mohamed, 2021 / Abraham, 2020 / Alaofe, 2021
		Assessing self-management of individuals.			The diabetes management self-efficacy scale (DMSES).		Saleh, 2021
	2nd group: mHealth guided self-management (e.g. Pain, blood pressure, blood glucose, medicine, foot care)	Monitoring via mobile applications/SMS.				*Facilitators: widely available smartphones.*	Farag Mohamed, 2021 /Pamungkas, 2022
	3rd group: Community-based support for self-management	Peer support / usage of existing services to support patients' families.		Community health workers (CHWs).	Healthcare professionals were on-site of the study to assess outcomes.	*Facilitators:* Community healthcare workers giving interactive training sessions /// Barriers: Countries lacking community healthcare workers.	Pienaar M., 2021 / Pamungkas,2021
Musculoskeletal Pain Conditions							
	1st group: Education	Tensile, strength, and hydrotherapy exercises.			Disease confirmed by orthopedic surgeon or radiologist before starting the intervention.	/// Barriers: Operating medical equipment / Buying equipment can be expensive.	Omidi, 2018 / Hendricks, 2022
		Weight loss and use of suitable footwear.					Hendricks, 2022
	2nd group: mHealth guided self-management (e.g. Pain, blood pressure, blood glucose, medicine, Foot care)						
	3rd group: Community-based support for self-management						
Hypertension	1st group: Education	Post discharge counselling / Online Public lectures / Interactive workshops / Educational sessions / low-salt, weight setting and diet education.	2 months (Adepu) / 1 month, 4 sessions per group (Khosravizade).	Pharmacists / Cardiology residents.	Direct questioning, group discussions, self-care practices or weight or blood pressure were measured at the start of educational program.	*Facilitators*: Easily understood educational methods / literacy ///*Barriers*: Costs.	Adepu, 2021 / Chu-Hong, 2015 / Khosravizade, 2015 / Voloshyna, 2018
		Assessing self-management of individuals.			Questionnaire	///*Barriers*: Results are limited by self-reported survey questions. There might be unobserved confounding factors that were not considered.	Qu, 2019
	2nd group: mHealth guided self-management (e.g. Pain, blood pressure, blood glucose, medicine, foot care)						
	3rd group: Community-based support for self-management						

Table S1. Table of characteristics of self-management interventions

Disease	Type of intervention	Details of intervention		Delivered by	Outcomes assessment	Facilitators/Barriers	Reference
		mode	*duration*				
Diabetes	1st group: Education	Focus groups / Interviews / Articles / education applications / Online consultations / Posters about diabetes / Face-to-Face education / Post discharge counselling / Self-care training and education course.	2 weeks; 10 minutes a session (Nazir) / 12 weeks; a session a week (Emara) / 6 Sessions (Guevara) / 4 sessions (Abraham) / 3 months (Thanh) / 2 months (Adepu).	Pharmacists / Family physicians / Nurses / Secondary school teachers with bachelor's degrees.	Pharmacists / Observed by physicians / Consultation by a diabetes care professional.	<u>*Facilitators:*</u> Face-to-face training by pharmacists / /// <u>*Barriers:*</u> Some pharmacists believe such interventions are outside the scope of conventional pharmacists' practice / community pharmacists might not be well-suited in all countries / *When interventions were assessed/restricted to a specific hospital, the data could not be generalized to the lifestyle and knowledge of all locals.*	Almomani, 2021 / Nazir, 2020 / Emara, 2021 / Pamungkas, 2022 / Abraham, 2020 / Thanh, 2021 / Adepu, 2021 / Alaofe, 2021 / Pamungkas, 2022 / Ahrari, 2021
		Focus groups / Interviews / Articles / education applications / Online consultations / Posters / Advice.					Almomani, 2021 / Rahmatullah, 2021 / Pamungkas, 2022 / Abraham, 2020
		Advice about smoking, nutrition, diet, and lifestyle.				<u>*Barriers:*</u> seasonality and distance were barriers to obtaining healthy food.	Almomani, 2021 / Pamungkas, 2022 / Farag Mohamed, 2021 / Abraham, 2020 / Alaofe, 2021
		Assessing self-management of individuals.			The diabetes management self-efficacy scale (DMSES).		Saleh, 2021
	2nd group: mHealth guided self-management (e.g. Pain, blood pressure, blood glucose, medicine, foot care)	Monitoring via mobile applications/SMS.				<u>*Facilitators*</u>*: widely available smartphones.*	Farag Mohamed, 2021 /Pamungkas, 2022
	3rd group: Community-based support for self-management	Peer support / usage of existing services to support patients' families.		Community health workers (CHWs).	Healthcare professionals were on-site of the study to assess outcomes.	<u>*Facilitators:*</u> Community healthcare workers giving interactive training sessions /// <u>Barriers: C</u>ountries lacking community healthcare workers.	Pienaar M., 2021 / Pamungkas,2021
Musculoskeletal Pain Conditions	1st group: Education						
		Tensile, strength, and hydrotherapy exercises.			Disease confirmed by orthopedic surgeon or radiologist before starting the intervention.	/// <u>Barriers:</u> Operating medical equipment / Buying equipment can be expensive.	Omidi, 2018 / Hendricks, 2022
		Weight loss and use of suitable footwear.					Hendricks, 2022
	2nd group: mHealth guided self-management (e.g. Pain, blood pressure, blood glucose, medicine, Foot care)						
	3rd group: Community-based support for self-management						
Hypertension	1st group: Education	Post discharge counselling / Online Public lectures / Interactive workshops / Educational sessions / low-salt, weight setting and diet education.	2 months (Adepu) / 1 month, 4 sessions per group (Khosravizade).	Pharmacists / Cardiology residents.	Direct questioning, group discussions, self-care practices or weight or blood pressure were measured at the start of educational program.	<u>*Facilitators*</u>: Easily understood educational methods / literacy ///<u>*Barriers*</u>: Costs.	Adepu, 2021 / Chu-Hong, 2015 / Khosravizade, 2015 / Voloshyna, 2018

		Assessing self-management of individuals.			Questionnaire	///*Barriers*: Results are limited by self-reported survey questions. There might be unobserved confounding factors that were not considered.	Qu, 2019
	2nd group: mHealth guided self-management (e.g. Pain, blood pressure, blood glucose, medicine, foot care)						
	3rd group: Community-based support for self-management						
